# Phimosis as a Cause of Rupture in Children

**Published:** 1878-09

**Authors:** 


Phimosis as a Cause of Rupture in Children.—Mr. J. Arthur Kempe reports, in the *Lancet*, a series of observations which leads him to believe that phimosis is not an unfrequent cause of hernia in infants. He states that the sequel of events is probably as follows: The abdominal parietes are naturally weak in children, which renders them less able to resist impulses which project the viscera against weakened parts. Here, then, is a remote or predisposing cause. The exciting cause is readily supplied by the frequent and continued efforts that the child makes to overcome the obstruction offered by the tight prepuce, and by the cries uttered consequent on pain caused in making these efforts.—*Med. and Surg. Reporter.*

